# Extracts of Selected South African Medicinal Plants Mitigate Virulence Factors in Multidrug-Resistant Strains of *Klebsiella pneumoniae*

**DOI:** 10.1155/2023/3146588

**Published:** 2023-10-14

**Authors:** Idowu J. Adeosun, Itumeleng T. Baloyi, Sekelwa Cosa

**Affiliations:** Division of Microbiology, Department of Biochemistry, Genetics and Microbiology, University of Pretoria, Private Bag X20, Hatfield Pretoria 0028, South Africa

## Abstract

The emergence of multidrug-resistant (MDR) *Klebsiella pneumoniae* remains a global health threat due to its alarming rates of becoming resistant to antibiotics. Therefore, identifying plant-based treatment options to target this pathogen's virulence factors is a priority. This study examined the antivirulence activities of twelve plant extracts obtained from three South African medicinal plants (*Lippia javanica, Carpobrotus dimidiatus*, and *Helichrysum populifolium*) against carbapenem-resistant (CBR) and extended-spectrum beta-lactamase (ESBL) positive *K. pneumoniae* strains. The plant extracts (ethyl acetate, dichloromethane, methanol, and water) were validated for their inhibitory activities against bacterial growth and virulence factors such as biofilm formation, exopolysaccharide (EPS) production, curli expression, and hypermucoviscosity. The potent extract on *K. pneumoniae* biofilm was observed with a scanning electron microscope (SEM), while exopolysaccharide topography and surface parameters were observed using atomic force microscopy (AFM). Chemical profiling of the potent extract *in vitro* was analysed using liquid chromatography-mass spectrometry (LC-MS). Results revealed a noteworthy minimum inhibitory concentration (MIC) value for the *C. dimidiatus* dichloromethane extract at 0.78 mg/mL on CBR- *K. pneumoniae*. *L. javanica* (ethyl acetate) showed the highest cell attachment inhibition (67.25%) for CBR- *K. pneumoniae.* SEM correlated the *in-vitro* findings, evidenced by a significant alteration of the biofilm architecture. The highest EPS reduction of 34.18% was also noted for *L. javanica* (ethyl acetate) and correlated by noticeable changes observed using AFM. *L. javanica* (ethyl acetate) further reduced hypermucoviscosity to the least length mucoid string (1 mm-2 mm) at 1.00 mg/mL on both strains. *C. dimidiatus* (aqueous) showed biofilm inhibition of 45.91% for the ESBL-positive *K. pneumoniae* and inhibited curli expression at 0.50 mg/mL in both *K. pneumoniae* strains as observed for *H. populifolium* (aqueous) extract. Chemical profiling of *L. javanica* (ethyl acetate), *C. dimidiatus* (aqueous), and *H. populifolium* (aqueous) identified diterpene (10.29%), hydroxy-dimethoxyflavone (10.24%), and 4,5-dicaffeoylquinic acid (13.41%), respectively, as dominant compounds. Overall, the ethyl acetate extract of *L. javanica* revealed potent antivirulence properties against the studied MDR *K. pneumoniae* strains. Hence, it is a promising medicinal plant that can be investigated further to develop alternative therapy for managing *K. pneumoniae*-associated infections.

## 1. Introduction

Plants have long been used as medicines to treat various ailments, and approximately, 100,000 plant species have been investigated for their medicinal purposes [1, 2]. This is based on medicinal plants possessing a diverse range of secondary metabolites such as tannins, terpenoids, alkaloids, and flavonoids, displaying antibacterial activities and suggesting their therapeutic properties [3]. As per the record, 80% of the emerging world's population relies on traditional medicine for therapy, while the World Health Organization (WHO) documents that ∼25% of all modern medicines were obtained from medicinal plants [4]. This further substantiates medicinal plants as a vital modern and traditional medicine source.

South Africans utilize traditional medicine to manage their physical, psychological, and primary healthcare needs [5]. Scott et al. [6] validate this by documenting that about 70% of South Africans use traditional medicines derived from plant species indigenous to the region. In addition, South Africa has ∼30,000 different plant species (∼10% of the world's higher plant species) [7]. These medicinal plants are, however, rarely explored as prospective drug candidates for the management of MDR pathogens, in particular, for their antivirulence properties. The pathogenic *Klebsiella pneumoniae* is listed among the most critical priority pathogens by WHO due to its resistance against almost all available conventional antibiotics [8].

*K. pneumoniae*, a Gram-negative, nonmotile, usually capsulated, facultatively anaerobic bacteria belonging to the family *Enterobacteriaceae* [9], is known for its pathogenicity towards humans causing digestive, urinary, and respiratory tract infections [10]. It has also been reported to be implicated with septicaemia, soft tissue infections, intraabdominal infections, and wound and blood infections [11]. *K. pneumoniae* possesses virulence factors such as capsular antigens, adherence factors for biofilm formation, *O*-lipopolysaccharide, exopolysaccharides, and siderophores linked to its infectivity and virulence [12, 13]. Combating its virulence has been challenging due to antibiotic resistance, which has increased dramatically over the last few decades [14], with resistance to *β*-lactams having the most significant impact on treatment efficacy [15]. Thus, new or alternative therapeutic options other than conventional antibiotics need to be studied to identify unorthodox methods to control antibiotic-resistant bacteria [16]. As a prospective solution to this global health threat, WHO recommends exploring medicinal plants for their antipathogenic or antivirulence potential as this might be the source of new drugs [17].

*Lippia javanica, Helichrysum populifolium*, and *Carpobrotus dimidiatus*, the three South African medicinal plants reported to possess high therapeutic potential against MDR bacteria might also play a role in the control of *K. pneumoniae* related infections [18, 19].

*Lippia javanica* (Burm.f.) Spreng commonly known as the fever tea belongs to the family *Verbenaceae* and has a long history of traditional uses in tropical Africa [18]. Based on its perceived medicinal characteristics, it is frequently used as an indigenous herbal tea, refreshing beverage, or as culinary addition [18]. *L. javanica* is rich in volatile oil, particularly ipsenone, carvone, ipsdienone, limonene, myrcene, ocimenone, myrcenone, piperitenone, linalool, sabinene, caryophyllene, tagetenone, *p*-cymene, among others [20], which are contributing factors to its medicinal properties.

*Helichrysum populifolium*, a member of the *Asteraceae* family, is commonly called poplar helichrysum [21]. In traditional medicine, plants belonging to the *Helichrysum* genus have a history of use in treating a wide range of conditions such as liver disorders, cystitis, jaundice, stomach pain, gall bladder complications, allergies, colds, cough, skin infections, asthma, inflammation, insomnia, arthritis, and for wound healing [22]. The health properties of this plant can be attributed to its bioactive compounds which include essential oils such as terpenoids [23], as well as flavonoids, phenolic acids, pyrone, benzofurans, and phloroglucinols [24].

*Carpobrotus dimidiatus* (Haw.) L. Bolus commonly known as natal sour fig is an indigenous South African species from the *Aizoaceae* family which grows abundantly in the east of the coastal regions in South Africa [25]. It is among the commercially important South African medicinal plants used in traditional medicine to treat skin infections, toothaches diabetes, wounds, sore throat, dysentery, high blood pressure, digestive ailments, and tuberculosis [26]. Bioactive compounds such as tannins, phytosterols, flavonoids, aromatic acids, and alkaloids are abundant in the *Carpobrotus* species [27].

Although the medicinal properties of these plants are well known, their antivirulence activities are yet to be explored. It is unknown if they contain antiinfectious phytochemicals for the management of *K. pneumoniae* infections. This study therefore aimed at studying the effect of *Lippia javanica, Helichrysum populifolium*, and *Carpobrotus dimidiatus* extracts on *K. pneumoniae* virulence.

## 2. Materials and Methods

### 2.1. Collection and Extraction of Plant Materials

Leaves of three plant species (*Lippia javanica, Helichrysum populifolium*, and *Carpobrotus dimidiatus*) were harvested from the Manie van der Schijff Botanical Garden, University of Pretoria. The identity of the plants was confirmed at the Department of Plant and Soil Sciences, University of Pretoria. Voucher specimen numbers were assigned as PRU 128530 for *Lippia javanica* PRU 128531 for *Helichrysum populifolium* and PRU 128529 for *Carpobrotus dimidiatus*) upon submission at the University of Pretoria H.G.W.J. Schweickerdt Herbarium.

Plant preparation and extractions were carried out according to the method used by Mashamba et al. [28]. The leaves were allowed to dry at room temperature (25°C) and were blended and weighed using a weighing balance (Kern 770, Microsep, Johannesburg, South Africa). Extraction was carried out using solvents of varying polarities, including dichloromethane, ethyl acetate, methanol, and water. Approximately, 30.00 g of each powdered plant material was extracted with 300 mL solvents of methanol and ethyl acetate, while for dichloromethane, 35.00 g of plant powder was used. The mixtures were shaken (Labcon, South Africa) at 140 rpm for 48 h. Afterwards, a Whatman no. 1 filter paper (11 µm) was used to filter the extracts. The filtrates were evaporated to dryness using a rotatory evaporator (Labotec Buchi Heidolph, Germany) at 45°C under reduced pressure and then dried entirely in a fume hood for 4-5 days.

For aqueous extraction, 300 mL of deionized water was added to 30.00 g of the blended plant material and allowed to boil at 100°C for 45 min on a hotplate (Labotec, South Africa). After cooling, the mixture was filtered using Whatman no. 1 filter paper (11 *μ*m), transferred to glass jars with screwcaps, frozen at −80°C for 3–6 h, and lyophilized (SP Scientific freeze dryer Scientific US, USA). Masses of the twelve dried plant extracts (*L. javanica* (aqueous), *L. javanica* (ethyl acetate), *L. javanica* (methanol), *L. javanica* (dichloromethane), *C. dimidiatus* (aqueous), *C. dimidiatus* (ethyl acetate), *C. dimidiatus* (methanol), *C. dimidiatus* (dichloromethane), *H. populifolium* (aqueous), *H. populifolium* (ethyl acetate), *H. populifolium* (methanol), and *H. populifolium* (dichloromethane) were determined. The extracts were stored at 4°C prior use for biological assays. Subsequently, the yield of the extracts was calculated and presented in percentages as follows (1):

(1) $$\begin{array}{c} \text{Percentage yield }\left(\%\right)=\frac{\text{dry crude extract}}{\text{dry initial material before extraction}}\times 100\text{.} \end{array}$$

### 2.2. Liquid Chromatography-Mass Spectrometry Analysis of Plant Extracts

The chemical constituents of the studied plants were determined using liquid chromatography-mass spectrometry (LC-MS). Compound separation was performed using a Waters Acquity Ultra Performance Liquid Chromatography (UPLC®) system (Waters Inc., USA) with ultrapure LC-grade water and acetonitrile (Romil-UpS™, Microsep, South Africa) acidified with 0.1% formic acid (99+% purity) (Thermo Scientific, South Africa). Compounds were eluted from a Luna® Omega (Part no: 00D-4752-AN, USA) 1.6 *μ*m C18 100 Å (2.1 mm ID × 100 mm length) column using a simple linear gradient of acetonitrile, e.g., 2–35% over 1–4 h followed by a fast ramp to a high organic concentration, e.g., 35–80% acetonitrile in 5 min, with an isocratic wash step using 100% acetonitrile for 1 min and a column reconditioning step with 97% water for 2 min. Volumes of 7.5 *μ*L were injected onto the column heated to 40°C, and the flow rate was set at 0.4 mL/min. The UPLC was coupled to a Waters® Synapt G2 high-definition quadrupole-time-of-flight (QTOF) mass spectrometer (Waters Inc., Milford, Massachusetts, USA) operated in the negative ionization mode. The ESI capillary voltage was 2.6 kV. The source temperature was set at 120°C, the sampling cone voltage at 25 V, the extraction cone voltage at 4.0 V, and the cone nitrogen flow at 20 L/h. The desolvation temperature was set at 350°C with a nitrogen flow of 600 L/h. Collision-induced fragmentation was performed at 4 V for the trap collision energy, and the transfer collision energy was ramped from 20 to 40 V. The instrument's mass axis was continually corrected by infusing 2 ng/*μ*L aqueous leucine enkephalin (*m*/*z* 555.2693). Mass spectral scans were collected every 0.3 seconds from 50 to 1 200 Da. MassLynx™ (version 4.1) software (Waters, USA) was used for data acquisition and analysis.

### 2.3. Bacterial Strains and Growth Conditions

Two strains of *K. pneumoniae* (ATCC BAA-1705)-CBR and (ATCC 700603)-ESBL producing were used in this study. Before usage, the strains were preserved as glycerol stocks at −80°C. These strains were then cultured in Mueller Hinton (MH) medium and incubated at 37°C to generate active bacterial cultures. To obtain an absorbance (OD_600nm_) of 0.10, a few colonies were dissolved in sterile distilled water and homogenized. The bacterial cell suspension was adjusted to obtain an equivalent of 0.5 McFarland standard. Ethics approval (reference number: NAS157/2021) for using the *K. pneumoniae* strains was granted by the Ethics Committee, Faculty of Natural and Agricultural Sciences, University of Pretoria.

### 2.4. Antibacterial Activity of Plant Extracts against *K. pneumoniae* Strains

Assessment of the minimum inhibitory concentration (MIC) of the plant extracts was carried out following the broth dilution method as described by Alves et al. [29]. Approximately, 1.00 mg/mL of the plant extracts were prepared as the stock concentration in 1% DMSO, and 100 *μ*L of MH broth was dispensed into the wells. Subsequently, 100 *μ*L of each plant extract, in triplicate, was introduced into the initial row of the microtiter plates.”

Following a series of dilutions in the A to H direction, decreasing values between 6.25 and 0.05 mg/mL were obtained. Approximately, 100 *μ*L of standardized bacterial strains (OD_600nm_ = 0.08–0.10) were then added to each well. Samples were incubated at 37°C for 24 h. Following incubation, 40 *μ*L of a 0.20 mg/mL solution of p-iodonitrotetrazolium violet (INT) was added to each well and further incubated at 37°C for 30 min. Clear wells with no colour change suggested inhibition of bacterial growth. Visual evaluation and recording of the MIC value for each plant extract were performed. The MIC was defined as the lowest concentration of plant extracts at which the test strain showed no visible growth.

### 2.5. In Vitro Reduction of Biofilm Formation

The studied plant extracts were assessed for their ability to inhibit biofilms at the initial cell attachment stage. Furthermore, the preformed biofilms (biomass measurement) and the fully established (mature) biofilm stages were also examined following the method described by Baloyi et al. [30] and Blando et al. [31]. The twelve plant extracts were tested against CBR and ESBL-producing *K. pneumoniae strains* for the three biofilm stages. Approximately, 100 µL of standardized bacterial suspension (OD_600nm_ = 0.10), 100 *μ*L of MH broth, and 100 *μ*L of the plant extracts were loaded into the wells for the initial cell attachment inhibition assay and were incubated at 37°C for 24 h. Quercetin and ciprofloxacin were used as positive controls.

For the preformed and mature biofilm experiments, 100 *μ*L of standardized bacterial suspension and 100 *μ*L of MH broth were loaded into the wells. The samples were incubated at 37°C for 8 h for preformed biofilm and 24 h for mature biofilm under static and dynamic conditions. After incubation, 100 *μ*L of the plant extracts were introduced into each well and further incubated for 24 h. Initial cell attachment, biofilm biomass, and mature biofilms were all examined using the modified crystal violet (CV) assay. The 96-well plates containing formed biofilms were rinsed using sterile distilled water to get rid of planktonic cells and media. The plates were oven dried at 60°C for 45 min. Afterwards, 1% CV solution was applied to the wells and incubated in the dark for 15 min. The wells were rinsed with sterile water to eliminate any residual stains. To enable semiquantitative assessment of biofilm formation, the wells were destained using 125 *µ*L of 95% ethanol. A new plate was subsequently coated with around 100 *µ*L of the destaining solution, and a multi-mode microplate reader (SpectraMax® paradigm) was used to measure the absorbance (OD_585nm_). The percentage inhibition was calculated using the following equation:

(2) $$\begin{array}{c} \text{Biofilm reduction }\left(\%\right)=\frac{\left({\text{Control}}_{585\text{nm}}-{\text{Test}}_{585\text{nm}}\right)}{\left({\text{Control}}_{585\text{nm}}\right)}\times 100\text{.} \end{array}$$

Interpretation of results was performed according to the criterion stated by Famuyide et al. [32]. Inhibitory activity was defined by values between 0 and 100%; however, it was further divided into three categories: ≥50% (good activity), 0 to 49% (weak activity), and negative values, which showed a rise in biofilm formation rather than its inhibition.

### 2.6. In Situ Visualization of Biofilms Using Scanning Electron Microscopy

To examine the density and morphology of *K. pneumoniae* biofilms, subinhibitory biofilm inhibitory concentrations of the most active plant extract were fixed and examined using a scanning electron microscope (SEM) following the method described by Wijesundara and Rupasinghe [33]. After promptly rinsing in PBS, biofilms were fixed (while remaining in a microtiter plate) for 2 h in 0.1 M sodium cacodylate buffer (pH 7.2) containing 2% glutaraldehyde. The biofilms were then washed with phosphate washing buffer three more times for 15 min each. Afterwards, they were subjected to a series of ethanol gradients at concentrations of 35%, 50%, 75%, 90%, and 100%, which resulted in the dehydration of the samples. All the gradient phases required exposure intervals of 15-min, and the treatment with 100% ethanol was performed three times. The samples were dried using a series of ethanol gradients (25 : 75, 50 : 50, 75 : 25, and 100 : 0) for 15 min each. The 100 : 0 dilution step was repeated thrice. Equal volume of hexamethyldisilane (HMDS) and 100% ethanol was added, and the samples were covered and let to stand for 1 h. The HMDS-ethanol mixture was removed, and new HMDS was promptly introduced. The plates were left to air-dry in a fume hood for 2 h. Afterwards, the biofilms were affixed to aluminum stubs, coated with a layer of gold-palladium (15 nm), and examined using a Zeiss crossbeam 540 scanning electron microscope.

### 2.7. Inhibition of Exopolysaccharide Production

The exopolysaccharide (EPS) reduction assay was conducted following the method previously described by Gopu and Shetty [34]. Approximately, 1% of *K. pneumoniae* was inoculated in sterile LB broth with and without plant extracts and incubated at 37°C for 24 h. LB broth was used for this assay as it enables rapid and high-yield growth for many species including *K. pneumoniae*. Biofilms stuck to the walls of the test tube containing the LB broth were collected to obtain crude exopolysaccharides. Centrifugation was conducted briefly at 5000*g* for 30 min at 2°C to remove late log phase cells. To precipitate the dislodged EPS, the supernatant was filtered and combined with three times its volume of cold ethanol and then left to incubate all night at 2°C. The resulting EPS precipitate was then separated by centrifugation at 8000*g* for 30 min, dissolved in 1 mL of deionized water, and stored at −40°C till it was needed. The quantity of EPS was determined by mixing 1 mL of the EPS solution with an equal volume of 5% phenol and 5 mL of concentrated sulfuric acid, resulting in the development of a red colour. To quantify the crude EPS, glucose served as a standard over a concentration range of 0.25 to 1.00 mg/mL and the *R*^2^ value was determined. The color's intensity was measured at 490 nm using a Biotek microplate reader.

### 2.8. Atomic Force Microscopy Assessment of Exopolysaccharide Inhibition

Atomic force microscopy was employed to examine the impact of the best plant extract (*L. javanica* - ethyl acetate) in revealing notable inhibition of exopolysaccharide in *K. pneumoniae* strains as previously described by Santana et al. [35]. The studied *K. pneumoniae* strains were cultured in LB media overnight, centrifuged at room temperature (2000*g*, 15 min), washed thrice in phosphate buffer (5 mM, pH 6.5), and approximately 10^8^ colony-forming units (CFU)/mL were resuspended into tubes containing the same buffer. Approximately, 100 *μ*L of the plant extracts (1.00 mg/mL) were introduced into 3 mL of the cell suspensions, followed by incubation at 37°C for a duration of 4 h. The controls contained no plant extract.

Following incubation, 1 mL samples from each treatment were obtained and then subjected to centrifugation at room temperature for 15 min at 6000*g*. A thin cell smear was subsequently prepared on a glass plate. The slides were left to air-dry and were examined using the Veeco Atomic Force Microscope (Dimension icon with ScanAsyst, Slovak Republic) at a scan frequency of about 300 kHz, a nominal constant of 32 Nm^−1^, a scan speed of 0.100 Hz, and a scan size of 5.00 *μ*m. Nanoscope Analysis ScanAsyst software (v 8.15, Slovak Republic) was used for the imaging analysis.

### 2.9. Reduction of Curli Expression

Twelve plant extracts were investigated for their effects on curli expression in *K. pneumoniae* strains following the method described by Hammar et al. [36]. For the preparation of the bacterial suspension, 100 *μ*L of *K. pneumoniae* strains (adjusted to optical density 0.1) and plant extracts were inoculated in 3 mL of LB broth and were incubated at 37°C for 24 h.

Approximately, 3 *μ*L of each bacterial suspension was then introduced onto brain heart infusion (BHI) agar plates enriched with sucrose and congo red (CRI) dye. Bacteria without curli fimbriae formed white colonies, indicating the absence of these structures. In contrast, *K. pneumoniae* that produced curli adhered to the congo red dye, resulting in red colonies. Plant extracts were not added to the control cultures.”

### 2.10. Hypermucoviscosity Reduction Assay

To ascertain the impact of the investigated plant extracts on the hypermucoviscosity of K. pneumoniae strains, the methodology described by Wiskur et al. [37] was employed. The pathogen was inoculated on BHI plates with the twelve plant extracts at different concentrations which ranged between 0.12 mg/mL and 1.00 mg/mL and thereafter incubated at 37°C for 24 h. A mucoviscous string was stretched from a single colony using a conventional bacteriological loop. Each *K. pneumoniae* strain was classified as mucoid or considered to have a hypermucoviscous phenotype upon the presence of string-like growth or a mucoid string measuring over 5 mm. The negative control cultures contained no plant extracts, while ciprofloxacin and quercetin were used as the positive controls.

### 2.11. Statistical Analysis

Mean standard deviations were computed using the Microsoft Excel Office (2016 version) for all data obtained from the independent experimental repeats in triplicates. Statistical differences were assessed with one-way analysis of variance (ANOVA) for the comparison of the mean differences in the inhibitory activities of extracts and controls using the SAS program (v. 9.4). Statistically significant difference was recorded for *ρ* values < 0.05.

## 3. Results

### 3.1. Plants Extract Yield

Leaves of *C. dimidiatus, H. populifolium,* and *L. javanica* extracted using four solvents of varying polarities (methanol, dichloromethane, ethyl acetate, and water) revealed different percentage yields as shown in Table 1. Methanol extracts of *C. dimidiatus* showed the highest yield (36.71%), followed by *L. javanica* (methanol) with a 19.88% yield. The lowest yield was obtained from the dichloromethane extract of *C. dimidiatus* (1.92%).

### 3.2. Liquid Chromatography-Mass Spectrometry Analysis of Selected Plant Extracts

LC-MS chemical profiling was carried out on the studied plants, namely, *L. javanica* (ethyl acetate), *C. dimidiatus* (aqueous), and *H. populifolium* (aqueous). Twenty-eight (28) compounds were identified from *L. javanica* (ethyl acetate) at different retention times, the mass-to-charge ratio (*m*/*z*), and peak intensities (Table 2). For *C. dimidiatus* (aqueous), 30 compounds were distinct; however, the identities of 28 were known, while two 2 were unknown (Table 3). Furthermore, sixteen (16) compounds were identified from *H. populifolium* (aqueous); however, two (2) were unknown (Table 4). Based on the mass spectrometry data analysis, different classes of phytochemical compounds were represented which included glucosides, flavonoids, quinic acids, and derivatives. Among them, 10.29% unknown diterpene, 10.24% hydroxy-dimethoxyflavone (flavonoid), and 13.41% 4,5-dicaffeoylquinic acid isomer (quinic acids and derivatives) were observed as the major constituents, showing the highest peak intensities in *L. javanica* (ethyl acetate), *C. dimidiatus* (aqueous), and *H. populifolium* (aqueous), respectively. Representative mass spectrometry chromatograms of the analysed extracts are illustrated in Figure S2 showing peaks that correspond to the data presented in Tables 2–4.

### 3.3. Minimum Inhibitory Concentration Determination of Plant Extracts on CBR and ESBL-Producing *K. pneumoniae* Strains

Antibacterial activities of twelve crude extracts against *K. pneumoniae* strains revealed MIC values ranging from 0.78 mg/mL to 6.25 mg/mL (Table 5). *C. dimidiatus* (dichloromethane) showed the best MIC value of 0.78 mg/mL on CBR-*K. pneumoniae* exhibiting inhibitory activity on bacterial growth. Other crude extracts tested showed varying MIC values for both strains (Table 5). *C. dimidiatus* (methanol) and dichloromethane extracts of *H. populifolium* and *L. javanica* showed higher MIC values of 6.25 mg/mL for CBR and ESBL-producing *K. pneumoniae* strains. The positive controls (quercetin and ciprofloxacin) showed noteworthy MIC activities with values of 0.06 mg/mL and 0.01 mg/mL, respectively.

### 3.4. Inhibition of Biofilm Formation

#### 3.4.1. Effect of Plant Extracts on Initial Cell Attachment

Antiadhesion (initial attachment) activity of plant extracts against CBR and ESBL-producing *K. pneumoniae* strains is shown in Table 6. The results revealed *L. javanica* (ethyl acetate) and *H. populifolium* (aqueous) as extracts with the highest cell attachment inhibitory activity (67.25%). Both showed good (above 50% inhibition) antiadhesion activity for CBR-*K. pneumoniae* similar to the result obtained for ciprofloxacin, while *C. dimidiatus* (aqueous) revealed an inhibitory activity of 45.91%, although the extract was the most prominent for ESBL-*K. pneumoniae* in comparison to the other extracts (Table 6).

The lowest level of activity against adhesion was revealed by *C. dimidiatus* (ethyl acetate extract) (0.07%) and *H. populifolium* (dichloromethane) (0.61%) for CBR-*K. pneumoniae* and ESBL-*K. pneumoniae*, respectively. No inhibition of initial cell attachment was observed for *H. populifolium* (methanol) and *H. populifolium* (ethyl acetate) for both strains of *K. pneumoniae* tested. Similarly, *L. javanica* (methanol) showed no cell attachment inhibition for CBR-*K. pneumoniae*. Ciprofloxacin exhibited potent inhibitory activity of 69.25% and 62.45% on the initial cell attachment of both strains tested (Table 6).

#### 3.4.2. Inhibition of Preformed Biofilms by Plant Extracts

Inhibition of preformed biofilm in the test strains upon the addition of the studied plant extracts was examined, and results are shown in Table 6. Most of the plant extracts showed a comparatively weaker ability to inhibit preformed biofilms, with no more than 45% biofilm inhibition in contrast to the initial attachment that had as high as 67%. The highest preformed biofilm inhibition by the plant extracts was shown by *L. javanica* (ethyl acetate) for both strains with 45.05% and 20.21% for CBR-*K. pneumoniae* and ESBL-*K. pneumoniae*, respectively (Table 6). This was relative to quercetin, which showed inhibitory activity of 35.15%, while ciprofloxacin was significantly higher, showing 71.42% inhibition for CBR-*K. pneumoniae*. For ESBL-*K. pneumoniae*, inhibition was recorded at 31.81% and 68.51% for quercetin and ciprofloxacin, respectively.

#### 3.4.3. Disruption of Mature Biofilm under Dynamic and Static Conditions

Under static and dynamic conditions, the impact of crude plant extracts on *K. pneumoniae* mature biofilms was assessed and findings are as presented in Table 7. *L. javanica* (aqueous) and *L. javanica* (ethyl acetate) demonstrated the inhibition of the mature biofilms of both strains under dynamic conditions, each at 20.79% for CBR-*K. pneumoniae* and 21.36% for ESBL-*K. pneumoniae,* respectively. However, under the same conditions, *L. javanica* (methanol), *C. dimidiatus* (ethyl acetate), *C. dimidiatus* (methanol), *H. populifolium* (aqueous), and *H. populifolium* (dichloromethane) did not show inhibition but rather enhanced the growth of mature biofilms. Ciprofloxacin inhibited the mature biofilm formed by CBR-*K. pneumoniae* at 42.16%. (ANOVA GLM, *F* = 2.29, DF = 12, *R*^2^ = 0.051, and *p* < 0.05). The mature biofilm inhibitory activity of ciprofloxacin was at 37.72% against ESBL-*K. pneumoniae* with differences found between the plant extracts and positive control (ANOVA GLM, *F* = 2.82, DF = 12, *R*^2^ = 0.074, and *p* < 0.05) (Table 7).

Similarly, for mature biofilms grown under static conditions, *L. javanica* (aqueous) also revealed the highest inhibitory activity on CBR-*K. pneumoniae* and ESBL-*K. pneumoniae*, at 16.45% and 11.73%, respectively (Table 7). The extracts showed weak or no inhibition of mature biofilms tested under static conditions. Only ciprofloxacin showed moderate inhibitory activity at 51.66% and 53.19% for CBR-*K. pneumoniae* and ESBL-*K. pneumoniae,* respectively. Statistical differences were observed in the mature biofilm inhibitory activity of ciprofloxacin when compared with the extracts (ANOVA GLM, *F* = 3.29, DF = 12, *R*^2^ = 0.068, and *p* < 0.05). Overall, lower inhibition was revealed by the extracts on matured biofilms formed under static conditions than the biofilms formed under dynamic conditions (Table 7).

#### 3.4.4. Scanning Electron Microscopy (SEM) Analysis of Biofilms

**Figure 1:**
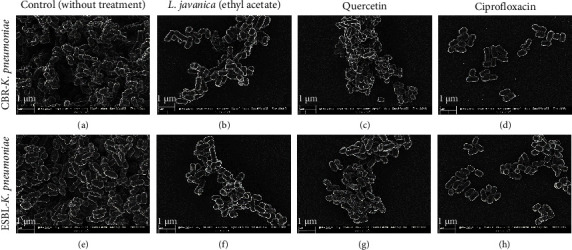
SEM micrographs showing biofilm inhibitory activity of *L. javanica* (ethyl acetate extract) against CBR and ESBL-producing *K. pneumoniae* at 20 KX magnification. (a) CBR-*K. pneumoniae* (without treatment), (b) CBR-*K. pneumoniae* (treated with *L. javanica* -ethyl acetate extract), (c) CBR-*K. pneumoniae* (treated with quercetin -positive control), (d) CBR-*K. pneumoniae* (treated with ciprofloxacin -positive control), (e) ESBL-*K. pneumoniae* (without treatment), (f) ESBL-*K. pneumoniae* (treated with *L. javanica* -ethyl acetate extract), (g) ESBL-*K. pneumoniae* (treated with quercetin-positive control), (h) ESBL-*K. pneumoniae* (treated with ciprofloxacin -positive control).

To gain a detailed view of the *K. pneumoniae* biofilms formed after subjection to treatment with the most efficient plant extract (*L. javanica* ethyl acetate), an analysis using SEM was conducted.  Figure 1 displays the SEM micrographs of the untreated biofilms (Figures 1(a) and 1(e)) and biofilms formed after treatment with *L. javanica* ethyl acetate extract (Figures 1(b) and 1(f)) and the positive controls: quercetin, 0.10 mg/mL (Figures 1(c) and 1(g)) and ciprofloxacin, 0.01 mg/mL (Figures 1(d) and 1(h)).

*L. javanica* (ethyl acetate) showed the best antibiofilm activity of all the studied plant extracts for CBR and ESBL-*K. pneumoniae*, as seen in Figures 1(b) and 1(f), respectively. The SEM micrographs revealed fewer clusters of connected microcolonies, indicating a considerable decrease in the number of biofilms. With very few clumps of dispersed cells, ciprofloxacin was found to have a more potent activity (Figures 1(d) and 1(h)).

Comparatively, the untreated biofilms showed a dense cluster of cells that exhibited continuous clumping and substantial collection of cells (Figures 1(a) and 1(e)). In comparison to *L. javanica* (ethyl acetate), quercetin was found to be less effective at disrupting the formed biofilms (Figures 1(c) and 1(g)); however, it displayed fewer cell clumps than the untreated biofilms.

#### 3.4.5. Reduction of *K. pneumoniae* Exopolysaccharides

The phenol-sulfuric acid technique was used to determine the quantity of EPS at the corresponding MIC values of test extracts in both pathogen strains. Results showed that there was good linearity, as evidenced by the correlation coefficient (*R*) value of 0.9419 (Figure S1).

**Figure 2:**
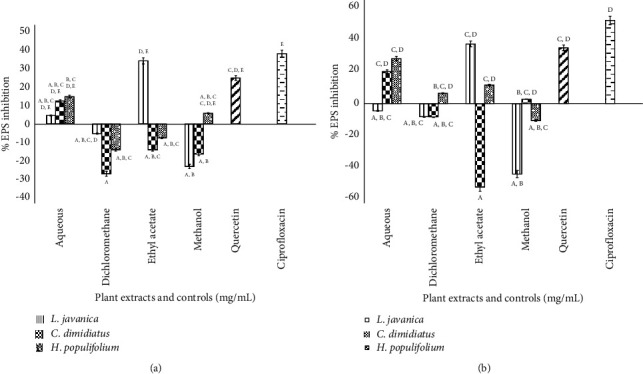
Exopolysaccharide percentage inhibition in ESBL-producing *K. pneumoniae* (a) and CBR- *K. pneumoniae*, (b) by all plant extracts at respective MIC values. Statistical significance of the test plant extracts and controls are indicated with different letters (A–E) with *p* value <0.05.

The most significant reduction in EPS produced by ESBL-*K. pneumoniae* was shown by *L. javanica* (ethyl acetate), which had the lowest EPS yield after treatment, resulting in 34.18% inhibition. On the other hand, the lowest EPS inhibition at 4.62% was revealed by *L. javanica* (aqueous). However, *L. javanica* (methanol), *L. javanica* (dichloromethane), *C. dimidiatus* (ethyl acetate), *C. dimidiatus* (methanol), *C. dimidiatus* (dichloromethane), *H. populifolium* (ethyl acetate), and *H. populifolium* (dichloromethane) revealed no inhibition of EPS, rather showing enhanced EPS production. Ciprofloxacin and quercetin revealed EPS inhibition of 38.11% and 24.94%, respectively (Figure 2(a)).

Furthermore, *L. javanica* (ethyl acetate) also revealed a moderate percentage of EPS inhibition (36.95%) for CBR-*K. pneumoniae*, followed by *H. populifolium* (aqueous) (27.85%). No EPS inhibitory activity was recorded for *L. javanica* (aqueous), *L. javanica* (methanol), *L. javanica* (dichloromethane), *C. dimidiatus* (ethyl acetate), *C. dimidiatus* (dichloromethane), and *H. populifolium* (methanol). Ciprofloxacin and quercetin revealed EPS inhibition at 51.56% and 34.55%, respectively, for the two *K. pneumoniae* strains (Figure 2(b)).

#### 3.4.6. Exopolysaccharides Microscopic Surface Topography Characterization

**Figure 3:**
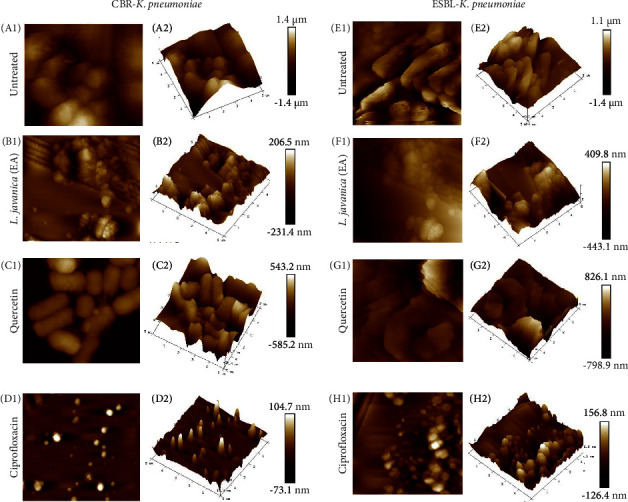
AFM micrographs showing the surface topography of exopolysaccharides produced by *Lippia javanica* (ethyl acetate extract) treated and untreated CBR and ESBL-producing *K. pneumoniae* strains at a scan size of 5.00 *μ*m (5,000 nm).

The surface topography of the examined *K. pneumoniae* exopolysaccharides (EPS) was captured using AFM. *L. javanica* (ethyl acetate) was selected for AFM due to the shown EPS inhibition (above) as compared to the other plant extracts examined. The AFM results revealed distinct variations between the topographies of EPS that had been treated and the untreated control. Untreated CBR and ESBL-producing *K. pneumoniae* produced EPS with irregular shapes and rough surfaces that were primarily made up of unevenly distributed lumps and were easily seen as foggy patches around the cells (Figure 3: A1 and E1). The EPS produced by both strains appeared tubular and compact when viewed with the microscope.

The treated EPS at MIC value showed the maximum lump heights of 206.5 nm and 409.8 nm for CBR and ESBL-producing *K. pneumoniae,* respectively (Figure 3: B1 and F1). The 3D scans revealed a marked decrease in surface roughness (Ra) (Figure 3: B2 and F2) where the Ra for CBR and ESBL-producing *K. pneumoniae* were 36.9 nm and 123 nm, respectively. The EPS subjected to treatment with positive control of ciprofloxacin also showed reduced surface roughness and height as revealed in Figure 3D and 3H.

#### 3.4.7. Curli Reduction in *K. pneumoniae* Strains

The effects of plant extracts on the presence of *K. pneumoniae* curli fibres are shown in Table 8. According to the findings, none of the plant extracts tested at concentrations of 0.12 mg/mL and 0.25 mg/mL inhibited the production of curli in the *K. pneumoniae* strains. Curli expression was reduced by *L. javanica* (ethyl acetate), *L. javanica* (dichloromethane), *C. dimidiatus* (aqueous), and *H. populifolium* (aqueous) extracts at 0.50 mg/mL in both strains. At the same concentration, *L. javanica* (aqueous) and *H. populifolium* (methanol) also inhibited the expression of curli in ESBL-*K. pneumoniae*. Furthermore, at 1.00 mg/mL, 50% of the plant extracts such as *L. javanica* (aqueous), *L. javanica* (ethyl acetate), *L. javanica* (dichloromethane), *C. dimidiatus* (aqueous), *H. populifolium* (aqueous), and *H. populifolium* (methanol) inhibited curli formation in both *K. pneumoniae* strains.

Ciprofloxacin reduced curli in the two strains tested at different concentrations (0.12 to 1.00 mg/mL), while quercetin only showed curli reduction at 0.50 and 1.00 mg/mL for both strains. No inhibitory activity was observed for the untreated control tested against both strains (Table 8).

#### 3.4.8. Reduction in Hypermucoviscosity Phenotype

**Figure 4:**
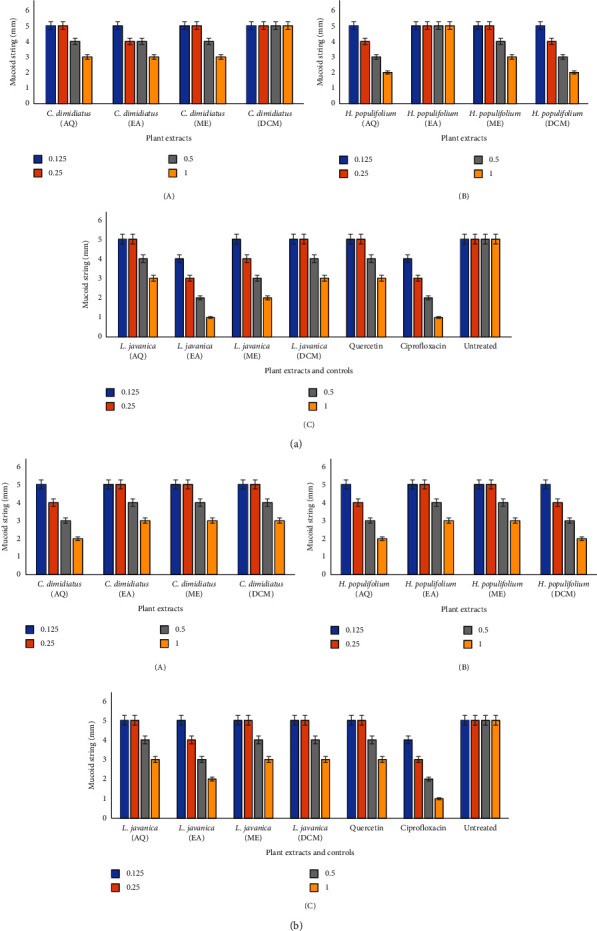
(a): Effect of plant extracts (*C. dimidiatus* (A), *H. populifolium* (B), and *L. javanica* (C)) in reducing CBR-*K. pneumoniae* hypermucoviscosity. Means are values of triplicate independent experiments ± SD. AQ: aqueous, EA: ethyl acetate, ME: methanol, and DCM: dichloromethane. (b) Effect of plant extracts (*C. dimidiatus* (A), *H. populifolium* (B), and *L. javanica* (C)) in inhibition of ESBL-*K. pneumoniae* hypermucoviscosity. Means are values of triplicate independent experiments ± SD. AQ: aqueous, EA: ethyl acetate, ME: methanol, and DCM: dichloromethane.

The impact of the studied plant extracts on *K. pneumoniae's* hypermucoviscosity was assessed through the use of a string test where the viscosity of the strains gradually decreased in a concentration dependent manner. For CBR-*K. pneumoniae* (Figure 4(a)), *L. javanica* (ethyl acetate) showed potent hypermucoviscosity reduction, observed by the least length of mucoid string (1 mm) at 1.00 mg/mL, followed by *L. javanica* (methanol), *H. populifolium* (aqueous), and *H. populifolium* (dichloromethane) (2 mm at 1.00 mg/mL). However, inhibition was not observed for *C. dimidiatus* (dichloromethane) and *H. populifolium* (ethyl acetate) at all the concentrations as observed with the negative control (Figure 4(a)-A, B, C).

Similarly, for ESBL-*K. pneumoniae* (Figure 4(b)), *L. javanica* (ethyl acetate), *C. dimidiatus* (aqueous), *H. populifolium* (aqueous), and *H. populifolium* (dichloromethane) revealed good hypermucoviscosity inhibition, showing the least length of mucoid string (2 mm) at 1.00 mg/mL and 3 mm at 0.50 mg/mL, while other plant extracts revealed the higher length of mucoid strings at those concentrations.

## 4. Discussion

Over the years, the use of plants in traditional medicine has piqued the attention of several researchers to discover effective plant extracts that can be used in the management of microbial infectious diseases [30]. South African medicinal plants are of great interest because despite the botanical and cultural diversity of South Africa, only a few plant species have hitherto become fully commercialised as medicinal products [38] and less explored for antivirulence activities. Therefore, three medicinal plants (*C. dimidiatus, H. populifolium,* and *L. javanica*) indigenous to South Africa, reported to have ethnomedicinal uses against *K. pneumoniae* infections, were examined in this study to validate some of their antipathogenic/antivirulence activities.

Since bioactive phytochemicals are vital and responsible for various activities, the extraction process is also crucial [39]. For this reason, varying extractants of different polarities were pivotal to deduce the most potent bioactive components from the plants. Results showed that the methanol extract had the highest yield followed by the aqueous extracts with up to 36.71% for *C. dimidiatus*. Congruent with our findings, Truong et al. [40], Itumeleng et al. [39], Adam et al. [41], and Eloff et al. [42] also reported a higher percentage yield from methanol extracts, followed by aqueous extracts. Highly polar solvents such as methanol and water thus favour extraction efficiency and yield compared to solvents with lesser polarity for plant species containing high levels of phenolic compounds.

For the development, modernisation, and quality control of various formulations from medicinal plants, chemical analysis of plant extracts is crucial [43]. Due to high sensitivity and accurate mass spectral detection, coupled with high-resolution chromatographic separation in LC-MS, analysis using this instrument has become more common in medicinal plant research [44]. For this reason, it was employed in our study for the chemical profiling of *L. javanica* (ethyl acetate), *C. dimidiatus* (aqueous), and *H. populifolium* (aqueous). The LC-MS analysis of the selected extracts revealed phytochemical compounds belonging to different classes such as the flavonoids, terpenes, verbascosides, phenolic acids, glycosides, quinic acids, and a derivative class of compounds which could contribute to their bioactivities. These classes of compounds are particularly interesting due to their previously reported pharmacological properties. For example, Maroyi [45] documented apigenin identified in *L. javanica* (Table 2) to possess antibacterial and hepatoprotective properties. Luteolin, another flavonoid shown in the spectra, has been reported by Kumar and Pandey [46] to possess anti-inflammatory and analgesic effects. According to Mohammad et al. [47], flavonoids, a group of naturally occurring phenolic compounds, are abundant in plants and are renowned for their significant health-promoting advantages. They have been documented to possess antibacterial, anti-inflammatory, antimutagenic, antiallergic, antithrombotic, and vasodilator properties [48].

Terpenes, also observed as part of the plants' compounds have been recognized as natural antimicrobial compounds [49]. Antimicrobial activities of terpenes against *Escherichia coli* O157: H7, *Salmonella typhimurium*, *Clostridium perfringens*, *Campylobacter jejuni*, and *Helicobacter pylori* have been reported by Mahizan et al. [50] and Thapa et al. [51]. Furthermore, Shi et al. [52] have reported the antibacterial activity of verbascosides against multidrug-resistant *P. aeruginosa*. This could be due to the presence of the unique multihydroxyl groups in their chemical structures which could perturb the lipid/water interface. Other possible mechanisms of inhibition by phytochemical compounds include competing and interfering with the activity of the signal molecules, due to their structural similarity or degradation of the signalling molecules [53].

The extracts (aqueous, ethyl acetate, methanol, and dichloromethane) from the three plant species were tested against two hypervirulent strains of *K. pneumoniae,* namely, carbapenem resistant (CBR) and extended spectrum beta lactamase (ESBL) *K. pneumoniae.* CBR-*K. pneumoniae* was investigated in this study due to its intriguing ability to produce carbapenemases (KPC), a common resistance mechanism seen in *K. pneumoniae*. This resistance mechanism poses a significant challenge for effective treatment, leading to a global health concern associated with high mortality rates [12]. Carbapenem-resistant *K. pneumoniae* exhibits rapid transmission, extensive resistance to antibiotics, and limited treatment options [12]. Similarly, ESBL-*K. pneumoniae* was studied due to its ability to produce extended-spectrum beta-lactamases (ESBL). According to Fils et al. [54], the worldwide spread of ESBL-*K. pneumoniae* is a critical issue, prompting the World Health Organization to categorize it as a priority pathogen, alongside other ESBL-producing *Enterobacteriaceae,* for which new treatment options are urgently needed [54].

The plant extracts at varying concentrations were assessed for their MIC activities against the studied CBR and ESBL *K. pneumoniae* strains. The MIC determination is considered the gold standard which reveals the lowest concentration of the treatment that inhibits the visible growth of the pathogen [55]. Findings from this study showed the MIC results that ranged from 0.78 mg/mL to 6.25 mg/mL. van Vuuren and Muhlarhi [56] defined MIC activities of plant extracts with noteworthy activities as values of 1.00 mg/mL or lower. Thus, *C. dimidiatus* (dichloromethane) was regarded as the most potent due to its MIC value of 0.78 mg/mL on CBR- *K. pneumoniae,* which was significantly lower than any of the other tested plant extracts. Several literature reports have been documented on the antibacterial properties of various plants against different pathogens such as in Mashamba et al. [28]. However, limited information exists on the antibacterial activity of *C. dimidiatus.* Variations observed in MIC values of the plant extracts may arise due to the difference in their chemical constituents [57]. The low activity of most of our tested plant extracts can be explained by the fact that *K. pneumoniae,* a Gram-negative bacterium, has a murein cell wall and an outer membrane, which is a complex barrier system against the permeation of polar plant extracts. Similar results have also been reported by Cosa et al. [7], Perera et al. [58], and Mogana et al. [59]. Furthermore, Gram-negative bacteria often reduce their outer membrane permeability by reducing the number of porins and inducing drug efflux pumps which transport drug molecules outwardly, making the bacterial cells resistant to treatments [60]. For these reasons, further studies using the plant extracts were focused on extracellular bacterial virulence factors of *K. pneumoniae*, including biofilm formation, exopolysaccharide, and curli production as well as hypermucoviscosity.

*K. pneumoniae* is known for its strong propensity to form biofilms which appear as a mucoid, cohesive slime layer, and it is considered a major factor in its resistance against antimicrobials and contributes to pathogenicity [61]. Biofilms are extracellular network-like aggregates of bacterial cells adhering to tissues, organs, and medical devices. They are composed of polysaccharides, extracellular DNA, and proteins [62]. The development of *K. pneumoniae* biofilm is initiated by the adhesion of cells [63], a proven preliminary stage which reveals an organism's potential to bind to the host cell [64], followed by the formation of microcolonies, maturation, and propagation of free-living cells [63]. The biofilm biomass screening in this study employed the crystal violet staining technique which has been reported by Ramos-Vivas et al. [65] to be widely accepted and used by many researchers due to the simplicity of its implementation for sessile biofilm detection.

The results showed that *L. javanica* (ethyl acetate) inhibited *K. pneumoniae* biofilm cell attachment, thereby reducing the pathogen's ability to attach and live in a protective scaffold. The ability of *L. javanica* extracts to reduce cell attachment is supported by the findings reported by Shirinda et al. [66] suggesting that the organic extract of *L. javanica* twigs inhibited the initial cell attachment of biofilm in *Clostridium perfringens.* Furthermore, *L. javanica* (ethyl acetate) also revealed the highest percentage inhibition on preformed and mature biofilm of *K. pneumoniae* strains. Based on the documented literature, this could be due to the activity of verbascoside, a prominent bioactive constituent identified in *L. javanica* as shown in Table 2. Shi et al. [52] have reported that verbascoside is implicated in the eradication of biofilms formed by Gram-negative bacteria such as *Pseudomonas aeruginosa* and *Staphylococcus aureus*. Congruent to this, findings from Jang et al. [67] also revealed over 60% inhibition of *E. coli* biofilms by phenylethanoid glycoside (verbascoside). Apigenin, also present in *L. javanica*, could be implicated in the antibiofilm activity observed. This can be supported by the findings of Liu et al. [68], where apigenin was shown to reduce the initial adherence and biofilms formed by *Streptococcus mutans.*

The weak inhibitory activities observed at this stage for the other studied plant extracts could be based on these extracts possessing phytochemicals serving as additional nutrients to promote bacterial growth [30]. Most of the plant extracts studied were observed to be less potent on the mature biofilms with not more than 21.36% biofilm reduction. Previous authors such as Bi et al. [69] and Mashamba et al. [28] had opined that eliminating preexisting biofilms by plant extracts poses a great challenge as several biofilm-forming bacteria have shown resistance. Once mature biofilms are formed, it is more difficult to treat and remove them [70]. This could be due to the complexity of the physical structure of mature biofilms which makes them difficult to eradicate. Other possible reasons for this can include the presence of persister cells, high volumes of exopolysaccharides, as well as phytochemical removal from the matrix by efflux pumps leading to a reduction in the bactericidal efficiency of administered treatments [70].

Overall, the antibiofilm experiments revealed that *L. javanica* (ethyl acetate) has the potential to disrupt *K. pneumoniae* cell aggregates before the biofilm fully forms. Based on the efficacy of this extract observed against *K. pneumoniae* biofilms, *in situ* visualization was employed for the qualitative observation of biofilm disruption using SEM due to its excellent resolution, magnification, and actual sample structure preservation [71, 72]. The SEM analysis confirmed the antibiofilm activity of *L. javanica* (ethyl acetate) against the studied *K. pneumoniae* strains. Furthermore, only few clumps of microcolonies, with a significant reduction in the number of biofilms compared to the untreated cells, were revealed.

A key component of the biofilm extracellular matrix often produced by a wide range of microorganisms is exopolysaccharide (EPS) [73]. EPS plays a major role in holding the bacterial community together, attaching the cells to solid surfaces, ensuring optimum hydration and availability of nutrients [74]. Since EPS aids *K. pneumoniae* immune invasion and increases pathogenicity in biofilm-forming organisms generally, we assessed EPS as a contributing factor to the pathogenicity of biofilm-forming *K. pneumoniae.* Out of all plant extracts tested for EPS inhibition, *L. javanica* (ethyl acetate) revealed the highest percentage of EPS inhibition. Little is known about the abilities of the studied plants to inhibit EPS production. However, extracts of *Mangifera indica* have been reported by Husain et al. [75] to decrease the production of EPS in a treated culture of *Pseudomonas aeruginosa* where the extract exhibited 50.20% and 58.30% reduction at 400 *μ*g/mL and 800 *μ*g/mL, respectively, which is a significantly higher activity than what we observed for *L. javanica* extracts.

To validate the inhibitory effect of *L. javanica* (ethyl acetate) on EPS production, *in situ* visualization was performed using the AFM. The AFM allows for the quantification of EPS, revealing their roughness and height at the nanometre scale [71, 76]. In this study, the AFM micrographs of EPS treated with *L. javanica* (ethyl acetate) at MIC value exhibited distinguishable changes in surface roughness and height observed when compared to the untreated EPS. This could be due to the presence of camphene, a bioactive constituent in *L. javanica* previously reported by Adeosun et al. [12] to show exopolysaccharide inhibitory activity against *K. pneumoniae*. The AFM-based methodology employed in this study was useful to provide surface information regarding the effect of plant extract on *K. pneumoniae* EPS, a major component that makes up its biofilm matrixosome.

Another factor increasing the pathogenicity in *K. pneumoniae* is its propensity to generate curli, known as thin aggregative fimbriae [77]. Curli is known for forming interbacterial bundles and interacting directly with the substratum, allowing for a cohesive and stable association of cell aggregates [78]. Hence, curli expression in bacteria is linked to biofilm formation, contributing to virulence [79]. A reduction in curli expression was observed for *L. javanica* (ethyl acetate), *L. javanica* (dichloromethane) as well as the aqueous extracts of *C. dimidiatus* and *H. populifolium* at 0.50 mg/mL for both *K. pneumoniae* strains. This infers that the plant extracts can efficiently inhibit the formation of curli, hence inhibiting their ability to adhere to host tissue and enhance biofilm formation. Bioactive compounds such as luteolin, tricin, and quercetin-rutinoside isomers 1 and 2 observed from the chemical profiling of *L. javanica* could have aided the curli reduction since Pruteanu et al. [80] have reported that the abovementioned compounds can inhibit the assembly of amyloid curli fibres and interfere with bacterial biofilm formation.

In addition to the aforementioned virulence factors, *K. pneumoniae's* hypermucoviscous nature contributes to its pathogenicity [81]. This factor is significant since it renders the hypervirulent *K. pneumoniae* strains resistant to macrophage phagocytosis and neutrophil-mediated death, allowing them to spread more efficiently throughout the body of the host [82]. A hypervirulent *K. pneumoniae* is frequently associated with a hypermucoviscous phenotype, a capsule-associated mucopolysaccharide web [83]. Results from this study revealed a gradual decrease in the viscosity of *K. pneumoniae* strains where *L. javanica* (ethyl acetate) was noted for a strong antihypermucoviscosity activity, with the least mucoid string length of less than 2 mm at 1.0 mg/mL concentration for the studied *K*. *pneumoniae* strains. This is significantly lower than the 5 mm standard length defined by a positive string test [84]. *L. javanica* (ethyl acetate) extracts thus revealed high potential in regulating the hypermucoviscosity phenotype.

The studied indigenous South African medicinal plants not only serve as abundant reservoirs of bioactive compounds but also possess antibacterial and antivirulence activities, suggesting that they are promising in the development of novel drugs to combat *K. pneumoniae* resistance.

As the persistence of multidrug resistance in *K. pneumoniae* continues thereby necessitating the need for alternative therapies [85], further studies may involve progressing through various drug development stages such as conducting *in-vivo* assays using unique cell lines, determining binding affinity, and other general screening to evaluate the biological and pharmacological activity of the potential drug candidates [86]. In addition, preclinical studies and clinical trials can be conducted. These phases encompass activities such as safety assessment, determining appropriate dosages, evaluating acute and chronic toxicity, stability, formulation components, pharmacokinetics, allergic responses, effectiveness, haemolytic and local irritation assessments, mutagenicity, reproductive and carcinogenic effects, and more. Subsequently, the ultimate step involves the Food and Drug Administration (FDA) filing, inspection, approval, and postmarket surveillance [86].

## 5. Conclusions

This study revealed the three selected South African medicinal plants (*C. dimidiatus, H. populifolium,* and *L. javanica*) as prospective antibacterial and antivirulent agents against CBR- and ESBL-positive *K. pneumoniae* strains. A notable antibacterial activity was observed for *C. dimidiatus* (dichloromethane). The plant of *L. javanica* decreased virulence factors of *K. pneumoniae* strains, indicating its potential to be used in the development of antipathogenic drugs. Chemical profiling of *L. javanica* (ethyl acetate), *C. dimidiatus* (aqueous), and *H. populifolium* (aqueous) identified diterpene, hydroxy-dimethoxyflavone, and 4,5-dicaffeoylquinic acid, respectively, as dominant compounds. The highly complex profile of chemical compounds from *C. dimidiatus, H. populifolium,* and *L. javanica* can be further explored for their antivirulence properties against *K. pneumoniae* strains. This study contributes to the search for solutions to the threats posed by antibiotic resistance through the exploration of plant extracts used in traditional medicine.

## Figures and Tables

**Table 1:** Crude extract yield (%) of studied medicinal plants after extraction with solvents of varying polarities.

Plant species	Family name	Common names	Voucher specimen number	Extract yield (%)			
				AQ	ME	DCM	EA
*Lippia javanica*	*Verbenaceae*	Fever tea, fever tree, lemon bush, wild sage, wild tea (E), beukesbos, beukesbossie (A)	128530	15.94	19.88	7.36	4.66
*Carpobrotus dimidiatus*	*Aizoaceae*	Natal sour fig (E), natalse suurvy/strandvy (A), ikhambi lamabulawo (Z)	128529	9.07	36.71	1.92	1.99
*Helichrysum populifolium*	*Asteraceae*	Poplar helichrysum (E), strooiblom (A)	128531	10.61	10.91	3.18	6.23

Key: E = English, A = afrikaans, *Z* = zulu, AQ = aqueous, ME = methanol, DCM = dichloromethane, and EA = ethyl acetate.

**Table 2:** LC-MS spectral analysis of *Lippia javanica* (ethyl acetate) extract.

Peak #	Retention time (mins)	*m*/*z*	Peak intensity (%)	Identities
1	145.22	461.17	1.56	Caffeoyl-rhamnosyl-glucoside
2	158.02	359.10	1.20	Hydroxy-dimethoxybenzoyl hexopyranose
3	198.20	387.17	2.29	Hydroxy-jasmonic acid-glucoside
4	206.47	419.12	4.29	Afzelechin-rhamnoside
5	221.04	389.11	2.02	Theveside
6	244.06	623.20	3.88	Verbascoside isomer 1
7	252.31	623.20	6.23	Verbascoside isomer 2
8	259.92	623.20	8.93	Verbascoside isomer 3
9	267.71	623.20	7.55	Verbascoside isomer 4
10	278.40	607.20	2.31	Luteolin-xylosyl-glucoside
11	289.26	637.21	3.00	Quercetin-rutinoside isomer 1
12	297.04	637.21	1.70	Quercetin-rutinoside isomer 2
13	310.33	665.21	1.37	Tetramethyl-quercetin-rutinoside
14	321.18	651.23	3.05	Matairresinol 4′[apiosyl-glucoside]
15	358.13	285.04	1.46	Luteolin
16	393.96	327.22	1.03	Possibly a diterpene
17	400.92	269.05	3.40	Apigenin
18	408.54	299.06	6.67	Diosmethin
19	416.80	329.07	2.94	Tricin
20	421.34	359.08	3.18	Trimethoxyflavone isomer 1
21	441.76	359.08	0.69	Trimethoxyflavone isomer 2
22	458.94	299.06	2.31	Chrysoeriol
23	470.45	313.07	8.24	Cirsimaritin
24	483.25	343.08	6.14	Eupatorin
25	499.78	373.09	0.85	Quercetagetin 3,5,6,3′-tetramethyl ether
26	511.94	283.06	1.80	Dihydroxy-methoxy-phenylcoumarin
27	522.80	313.07	1.60	Kaempferol-dimethylether
28	539.64	501.32	10.29	Unknown diterpene

**Table 3:** LC-MS spectral analysis of *Carpobrotus dimidiatus* (aqueous) extract.

Peak #	Retention time (mins)	*m*/*z*	Peak intensity (%)	Identities
1	144.58	153.02	1.89	Dihydroxybenzoic acid
2	151.54	203.08	3.50	Unknown
3	175.85	337.09	6.39	Coumaroyl quinic acid isomer 1
4	192.38	367.10	1.57	Feruloyl quinic acid
5	202.60	337.09	7.92	Coumaroyl quinic acid isomer 2
6	209.56	337.09	6.81	Coumaroyl quinic acid isomer 3
7	222.37	297.06	10.24	Hydroxy-dimethoxyflavone
8	233.87	813.17	2.41	Luteolin triglucoside
9	239.54	683.14	1.72	Trihydroxy-trimethoxy flavone-diglucoside
10	250.40	797.18	2.08	Apigenin-feruloyl-diglucoside
11	259.31	799.23	4.13	Similar to tricin rutinoside-glucoside
12	262.24	785.21	1.49	Similar to isorhamnetin rutinoside-glucoside
13	267.75	653.17	2.96	Similar to syringentin-3-rutinoside
14	279.08	653.17	4.45	Syringentin-rutinoside-like
15	297.08	797.21	3.85	Similar to kaempferol-diglucoside-acetorhamnosyl
16	302.11	651.15	4.17	Similar to kaempferol-acetylglucosyl-glucoside
17	316.85	765.19	3.68	Could be an acetylated pinosylvin-diglucoside
18	323.18	795.20	5.67	Could be catechin-gallate-glucoside-glucuronide
19	330.14	765.19	0.62	Could be another isomer acetylated pinosylvin-diglucoside
20	339.05	619.13	2.34	Similar to apigenin acetylcoumaroyl glucoside
21	346.84	649.14	4.72	Similar to pelargonidin-xylosyl-malonyl-glucoside
22	359.47	331.04	0.84	Similar to quercetaggetin-3′-methyl-ether
23	368.39	256.10	0.66	Unknown nitrogen-containing compound
24	373.57	649.14	0.70	Similar to pelargonidin-xylosyl-malonyl-glucoside
25	393.34	327.22	6.74	Could be an oxygenated diterpene
26	421.38	329.23	3.35	Could be an oxygenated diterpene
27	470.48	313.07	0.92	Similar to kaempferol-dimethyl-ether
28	483.28	343.08	0.88	Dihydroxy-3-methoxyflavonone
29	513.27	293.18	2.28	Unknown
30	524.78	237.11	1.00	Methoxychalcone or cinnamyl-benzoate

**Table 4:** LC-MS spectral analysis of *Helichrysum populifolium* (aqueous) extract.

Peak #	Retention time (mins)	*m*/*z*	Peak intensity (%)	Identities
1	146.58	353.09	2.90	Chlorogenic acid isomer 1
2	166.35	293.12	5.40	Ethyl 3-hydroxybuterate glucoside
3	181.58	353.09	10.80	Chlorogenic acid isomer 2
4	200.70	353.09	5.66	Chlorogenic acid isomer 3
5	218.04	515.12	5.71	4,5-Dicaffeoylquinic acid isomer 1
6	231.98	367.10	2.01	Feruloylquinic acid
7	235.87	463.09	3.73	Hydroxykaempferol glucoside isomer 1
8	243.49	213.12	4.68	Unknown
9	253.69	567.21	2.23	Similar to citrusin B
10	257.59	463.09	5.78	Hydroxykaempferol glucoside isomer 2
11	268.93	515.12	6.03	4,5-Dicaffeoylquinic acid isomer 2
12	276.07	415.20	2.80	Unknown
13	280.44	515.12	13.41	4,5-Dicaffeoylquinic acid isomer 3
14	287.57	515.12	8.29	4,5-Dicaffeoylquinic acid isomer 4
15	332.79	491.12	2.73	Similar to lagotiside
16	349.96	677.15	3.67	Pelargonidin di-acetylglucoside
17	393.40	327.22	7.50	Possibly a diterpene
18	422.74	329.23	6.68	Tricin

**Table 5:** Minimum inhibitory concentration values (mg/mL) of plant extracts tested against *K. pneumoniae* strains.

Plant extracts	*K. pneumoniae* strains and MIC (mg/mL) values	
	CBR-*K. pneumoniae*	ESBL-*K. pneumoniae*
Aqueous extracts		
*L. javanica*	1.56	1.56
*C. dimidiatus*	6.25	3.12
*H. populifolium*	1.56	3.12
Dichloromethane extracts		
*L. javanica*	6.25	6.25
*C. dimidiatus*	0.78	3.12
*H. populifolium*	6.25	6.25
Ethyl acetate extracts		
*L. javanica*	3.12	1.56
*C. dimidiatus*	3.12	3.12
*H. populifolium*	3.12	6.25
Methanol extracts		
*L. javanica*	6.25	1.56
*C. dimidiatus*	6.25	6.25
*H. populifolium*	3.12	1.56

Controls		

Quercetin	0.06	0.06
Ciprofloxacin	0.01	0.01
1% DMSO	6.25	6.25

The MIC values are presented as the mean values of triplicates.

**Table 6:** Effect of plant extracts on initial cell attachment and biofilm development of *K. pneumoniae* strains.

Plant extracts and control	Percentage (%) inhibition of initial cell attachment		Percentage (%) inhibition of biofilm development	
	CBR-*K. pneumoniae*	ESBL- *K. pneumoniae*	CBR-*K. pneumoniae*	ESBL-*K. pneumoniae*
Aqueous extracts				
*L. javanica*	49.40 ± 0.04^c,d^	15.17 ± 0.05^a,b,c,d^	42.37 ± 0.07^c^	13.87 ± 0.01^d,e^
*C. dimidiatus*	48.11 ± 0.05^c,d^	45.91 ± 0.02^d^	11.31 ± 0.03^b,c^	−45.47 ± 0.01^a,b,c^
*H. populifolium*	67.25 ± 0.06^d^	14.04 ± 0.05^a,b,c,d^	12.80 ± 0.02^b,c^	8.25 ± 0.04^c,d,e^
Dichloromethane extracts				
*L. javanica*	34.13 ± 0.02^b,c,d^	28.52 ± 0.04^b,c,d^	−7.11 ± 0.08^a,b,c^	8.31 ± 0.02^c,d,e^
*C. dimidiatus*	22.81 ± 0.09^b,c,d^	23.80 ± 0.03^b,c,d^	11.16 ± 0.07^a,b^	12.75 ± 0.11^b,c,d^
*H. populifolium*	3.33 ± 0.04^a,b,c^	0.61 ± 0.04^a,b,c^	−60.29 ± 0.03^a^	−26.85 ± 0.06^a,b,c,d^
Ethyl acetate extracts				
*L. javanica*	67.25 ± 0.01^d^	28.77 ± 0.10^b,c,d^	45.05 ± 0.08^b,c^	20.21 ± 0.01^d,e^
*C. dimidiatus*	0.07 ± 0.01^a,b,c^	20.21 ± 0.06^b,c,d^	9.72 ± 0.05^b,c^	−59.11 ± 0.14^a,b^
*H. populifolium*	−20.58 ± 0.07^a,b^	−7.93 ± 0.09^a,b^	−4.02 ± 0.04^a,b,c^	1.56 ± 0.14^c,d,e^
Methanol extracts				
*L. javanica*	−50.31 ± 0.11^a^	12.57 ± 0.02^b,c,d^	−27.80 ± 0.02^a,b^	4.64 ± 0.03^c,d,e^
*C. dimidiatus*	40.45 ± 0.07^c,d^	37.41 ± 0.02^c,d^	−17.98 ± 0.17^a,b,c^	−39.89 ± 0.05^a,b,c,d^
*H. populifolium*	−19.62 ± 0.05^a,b^	−35.26 ± 0.05^a^	35.15 ± 0.03^b,c^	19.83 ± 0.04^a^

Controls				

Quercetin	42.57 ± 0.03^c,d^	40.66 ± 0.01^b,c^	35.15 ± 0.01^c,d^	31.81 ± 0.02^a,b^
Ciprofloxacin	69.25 ± 0.03^d^	62.45 ± 0.04^e^	71.42 ± 0.03^b,c^	68.51 ± 0.02^e^

The presented values represent the average from three separate and independent experiments, alongside the standard deviation (SD). Comparison of percentage inhibition at MIC value per K. pneumoniae strain was performed across each treatment. Different letters (a–e) indicate significant differences at *p* < 0.05 between the different treatments (against all extracts) at the same MIC value.

**Table 7:** Effect of plant extracts on disruption of mature biofilms formed by *K. pneumoniae* under dynamic and static conditions.

Plant extracts and control	Percentage (%) inhibition of mature biofilm formed under dynamic condition		Percentage (%) inhibition of mature biofilm formed under static condition	
	CBR-*K. pneumoniae*	ESBL-*K. pneumoniae*	CBR-*K. pneumoniae*	ESBL-*K. pneumoniae*
Aqueous extracts				
*L. javanica*	20.79 ± 0.01^a,b^	15.90 ± 0.01^a,b^	16.45 ± 0.01^b^	11.73 ± 0.02^a^
*C. dimidiatus*	13.01 ± 0.01^a,b^	−55.83 ± 0.01^a,b^	−25.77 ± 0.03^a,b^	−37.11 ± 0.04^a^
*H. populifolium*	−1.48 ± 0.04^a,b^	6.51 ± 0.02^a,b^	−19.89 ± 0.02^a,b^	7.52 ± 0.01^a^
Dichloromethane extracts				
*L. javanica*	17.35 ± 0.03^a,b^	1.12 ± 0.01^a,b^	−14.13 ± 0.03^a,b^	3.63 ± 0.01^a^
*C. dimidiatus*	7.57 ± 0.01^a,b^	−2.37 ± 0.01^a,b^	11.54 ± 0.02^a,b^	9.63 ± 0.01^a^
*H. populifolium*	−10.19 ± 0.02^a,b^	−37.67 ± 0.01^a,b^	−37.45 ± 0.04^a,b^	−32.79 ± 0.01^a^
Ethyl acetate extracts				
*L. javanica*	8.69 ± 0.02^a,b^	21.36 ± 0.02^a,b^	6.88 ± 0.07^a,b^	7.28 ± 0.02^a^
*C. dimidiatus*	−20.20 ± 0.12^a^	−89.23 ± 0.20^a^	−61.64 ± 0.15^a,b^	−73.14 ± 0.21^a^
*H. populifolium*	9.39 ± 0.04^a,b^	11.18 ± 0.03^a,b^	−61.34 ± 0.06^a,b^	−91.55 ± 0.24^a^
Methanol extracts				
*L. javanica*	−7.41 ± 0.01^a,b^	−26.83 ± 0.01^a,b^	−46.40 ± 0.05^a,b^	−18.52 ± 0.02^a^
*C. dimidiatus*	−21.99 ± 0.02^a^	−66.48 ± 0.03^a,b^	−86.72 ± 0.04^a^	−67.79 ± 0.05^a^
*H. populifolium*	17.17 ± 0.01^a,b^	−15.73 ± 0.02^a,b^	11.81 ± 0.02^a,b^	−84.85 ± 0.26^a^

Controls				

Quercetin	−27.08 ± 0.01^a^	−44.55 ± 0.01^a^	−35.46 ± 0.02^a^	−52.25 ± 0.02^a^
Ciprofloxacin	42.16 ± 0.01^b^	37.72 ± 0.02^b^	51.66 ± 0.01^c^	53.19 ± 0.01^b^

The presented values represent the average from three separate and independent experiments, alongside the standard deviation (SD). Comparison of percentage inhibition at MIC value per K. pneumoniae strain was performed across each treatment. Different letters (a–c) indicate significant differences at *p* < 0.05 between the different treatments (against all extracts) at the same MIC value.

**Table 8:** Effect of plant extracts on *Klebsiella pneumoniae *curli.

Plant extracts	Concentration (mg/mL) (A)					Concentration (mg/mL) (B)				
	Control	0.12	0.25	0.50	1.00	Control	0.12	0.25	0.50	1.00
Aqueous extracts										
*L. javanica*	+	+	+	+	−	+	+	+	−	−
*C. dimidiatus*	+	+	+	−	−	+	+	+	−	−
*H. populifolium*	+	+	+	−	−	+	+	+	−	−
Dichloromethane extracts										
*L. javanica*	+	+	+	−	−	+	+	+	−	−
*C. dimidiatus*	+	+	+	+	+	+	+	+	+	+
*H. populifolium*	+	+	+	+	+	+	+	+	+	+
Ethyl acetate extracts										
*L. javanica*	+	+	+	−	−	+	+	+	−	−
*C. dimidiatus*	+	+	+	+	+	+	+	+	+	−
*H. populifolium*	+	+	+	+	+	+	+	+	+	+
Methanol extracts										
*L. javanica*	+	+	+	+	+	+	+	+	+	+
*C. dimidiatus*	+	+	+	+	+	+	+	+	+	+
*H. populifolium*	+	+	+	+	−	+	+	+	−	−

Controls										

Quercetin	+	+	+	−	−	+	+	+	−	−
Ciprofloxacin	+	−	−	−	−	+	−	−	−	−
Untreated	+	+	+	+	+	+	+	+	+	+

Key: A: CBR-*K. pneumoniae,* B: ESBL-*K. pneumoniae,* +: positive, −: negative, control: untreated *K. pneumoniae* strains.

## Data Availability

All data and supplementary data is included in the manuscript.

## Supplementary Materials

Supplementary Materials The following supplementary data form part of this manuscript which further provides relevant information on some of the findings obtained from this study. Figure S1 shows a standard curve which entails the regression equation (*Y* = 0.348*X* − 0.074) obtained for exopolysaccharide quantification. Here, Y stands for the absorbance derived from the unknown samples. Figure S2 illustrates the representative mass spectrometry chromatograms of the analysed plant extracts showing peaks that correspond to the data presented in Tables 2–4.

## References

[1] Uc-Cachón A. H., Dzul-Beh A. J., Palma-Pech G. A. (2021). Antibacterial and antibiofilm activities of Mayan medicinal plants against Methicillin-susceptible and resistant strains of *Staphylococcus aureus*. *Journal of Ethnopharmacology*.

[2] Górniak I., Bartoszewski R., Króliczewski J. (2019). Comprehensive review of antimicrobial activities of plant flavonoids. *Phytochemistry Reviews*.

[3] Atanasov A. G., Zotchev S. B., Dirsch V. M. (2021). Natural products in drug discovery: advances and opportunities. *Nature Reviews Drug Discovery*.

[4] Chintamunnee V., Mahomoodally M. F. (2012). Herbal medicine commonly used against non-communicable diseases in the tropical island of Mauritius. *Journal of Herbal Medicine*.

[5] Agbor A. M., Naidoo S. (2016). A review of the role of African traditional medicine in the management of oral diseases. *African Journal of Traditional, Complementary and Alternative Medicines*.

[6] Scott G., Springfield E. P., Coldrey N. (2004). A pharmacognostical study of 26 South African plant species used as traditional medicines. *Pharmaceutical Biology*.

[7] Cosa S., Rakoma J. R., Yusuf A. A., Tshikalange T. E. (2020). *Calpurnia aurea* (Aiton) benth extracts reduce quorum sensing controlled virulence factors in *Pseudomonas aeruginosa*. *Molecules*.

[8] Boucher H. W., Talbot G. H., Bradley J. S. (2009). Bad bugs, no drugs: No ESKAPE! An update from the Infectious Diseases Society of America. *Clinical Infectious Diseases*.

[9] Kendaganna P. H., Shivamallu C., Shruthi G. (2021). *In silico* screening and validation of KPHS_00890 protein of *Klebsiella pneumoniae* proteome: an application to bacterial resistance and pathogenesis. *Journal of King Saud University Science*.

[10] Brisse S., Fevre C., Passet V. (2009). Virulent clones of *Klebsiella pneumoniae*: identification and evolutionary scenario based on genomic and phenotypic characterization. *PLoS One*.

[11] Adeosun I. J., Baloyi I., Aljoundi A. K., Salifu E. Y., Ibrahim M. A., Cosa S. (2022). Molecular modelling of SdiA protein by selected flavonoid and terpenes compounds to attenuate virulence in *Klebsiella pneumoniae*. *Journal of Biomolecular Structure and Dynamics*.

[12] Adeosun I. J., Baloyi I. T., Cosa S. (2022). Anti-biofilm and associated anti-virulence activities of selected phytochemical compounds against *Klebsiella pneumoniae*. *Plants*.

[13] Wareth G., Neubauer H. (2021). The Animal-foods-environment interface of *Klebsiella pneumoniae* in Germany: an observational study on pathogenicity, resistance development and the current situation. *Veterinary Research*.

[14] Adeosun I. J., Oladipo K. E., Ajibade O. A. (2019). Antibiotic susceptibility of *Klebsiella pneumoniae* isolated from selected tertiary hospitals in Osun state, Nigeria. *Iraqi Journal of Science*.

[15] Martin R. M., Bachman M. A. (2018). Colonization, infection, and the accessory genome of *Klebsiella pneumoniae*. *Frontiers in Cellular and Infection Microbiology*.

[16] Bouyahya A., Dakka N., Et-Touys A., Abrini J., Bakri Y. (2017). Medicinal plant products targeting quorum sensing for combating bacterial infections. *Asian Pacific Journal of Tropical Medicine*.

[17] Doughari J. H., Human I. S., Bennade S., Ndakidemi P. A. (2009). Phytochemicals as chemotherapeutic agents and antioxidants: possible solution to the control of antibiotic resistant verocytotoxin producing bacteria. *Journal of Medicinal Plants Research*.

[18] Chagonda L. S., Chalchat J. C. (2015). Essential oil composition of *Lippia javanica* (Burm.f.) spreng chemotype from Western Zimbabwe. *Journal of Essential Oil Bearing Plants*.

[19] Akinyede K. A., Ekpo O. E., Oguntibeju O. O. (2020). Ethnopharmacology, therapeutic properties and nutritional potentials of *Carpobrotus edulis*: a comprehensive review. *Scientia Pharmaceutica*.

[20] Nzira L., Per M., Peter F., Claus B. (2009). *Lippia javanica* (Burm F) Spreng: its general constituents and bioactivity on mosquitoes. *Tropical Biomedicine*.

[21] Lourens A. C. U., Viljoen A. M., van Heerden F. R. (2008). South African *Helichrysum* species: a review of the traditional uses, biological activity and phytochemistry. *Journal of Ethnopharmacology*.

[22] Akinyede K. A., Cupido C. N., Hughes G. D., Oguntibeju O. O., Ekpo O. E. (2021). Medicinal properties and *in vitro* biological activities of selected *Helichrysum* species from South Africa: a review. *Plants*.

[23] Mari A., Napolitano A., Masullo M., Pizza C., Piacente S. (2014). Identification and quantitative determination of the polar constituents in *Helichrysum italicum* flowers and derived food supplements. *Journal of Pharmaceutical and Biomedical Analysis*.

[24] Akaberi M., Sahebkar A., Azizi N., Emami S. A. (2019). Everlasting flowers: phytochemistry and pharmacology of the genus *Helichrysum*. *Industrial Crops and Products*.

[25] Broomhead N. K., Moodley R., Jonnalagadda S. B. (2020). Elemental analysis of the edible fruit of Carpobrotus dimidiatus (from Kwazulu-Natal, South Africa) and the influence of soil quality on its elemental uptake. *Journal of Environmental Science and Health, Part B*.

[26] Mulaudzi R. B., Aremu A. O., Rengasamy K. R. (2019). Antidiabetic, anti-inflammatory, anticholinesterase and cytotoxicity determination of two *Carpobrotus* species. *South African Journal of Botany*.

[27] Springfield E. P., Amabeoku G., Weitz F., Mabusela W., Johnson Q. (2003). An assessment of two *Carpobrotus* species extracts as potential antimicrobial agents. *Phytomedicine*.

[28] Mashamba T. G., Adeosun I. J., Baloyi I. T., Tshikalange E. T., Cosa S. (2022). Quorum sensing modulation and inhibition in biofilm forming foot ulcer pathogens by selected medicinal plants. *Heliyon*.

[29] Alves M. J., Ferreira I. C. F. R., Froufe H. J. C., Abreu R. M. V., Martins A., Pintado M. (2013). Antimicrobial activity of phenolic compounds identified in wild mushrooms, SAR analysis and docking studies. *Journal of Applied Microbiology*.

[30] Baloyi I. T., Adeosun I. J., Yusuf A. A., Cosa S. (2021). *In silico* and *in vitro* screening of antipathogenic properties of *Melianthus comosus* (Vahl) against *Pseudomonas aeruginosa*. *Antibiotics*.

[31] Blando F., Russo R., Negro C., De Bellis L., Frassinetti S. (2019). Antimicrobial and antibiofilm activity against *Staphylococcus aureus* of *Opuntia ficus-indica* (L.) mill. cladode polyphenolic extracts. *Antioxidants*.

[32] Famuyide I. M., Aro A. O., Fasina F. O., Eloff J. N., McGaw L. J. (2019). Antibacterial and antibiofilm activity of acetone leaf extracts of nine under-investigated south African *Eugenia* and *Syzygium* (*Myrtaceae*) species and their selectivity indices. *BMC Complementary and Alternative Medicine*.

[33] Wijesundara N. M., Rupasinghe H. (2019). Bactericidal and anti-biofilm activity of ethanol extracts derived from selected medicinal plants against *Streptococcus pyogenes*. *Molecules*.

[34] Gopu V., Shetty P. H. (2016). Cyanidin inhibits quorum signalling pathway of a food borne opportunistic pathogen. *Journal of Food Science and Technology*.

[35] Santana H. F., Barbosa A. A. T., Ferreira S. O., Mantovani H. C. (2012). Bactericidal activity of ethanolic extracts of propolis against *Staphylococcus aureus* isolated from mastitic cows. *World Journal of Microbiology and Biotechnology*.

[36] Hammar M., Arnqvist A., Bian Z., Olsén A., Normark S. (1995). Expression of two csg operons is required for production of fibronectin- and Congo red-binding curli polymers in *Escherichia coli* K-12. *Molecular Microbiology*.

[37] Wiskur B. J., Hunt J. J., Callegan M. C. (2008). Hypermucoviscosity as a virulence factor in experimental *Klebsiella pneumoniae* endophthalmitis. *Investigative Opthalmology and Visual Science*.

[38] Van Wyk B. E. (2011). The potential of South African plants in the development of new medicinal products. *South African Journal of Botany*.

[39] Itumeleng T. B., Idowu J. A., Abdullahi A. Y., Sekelwa C. (2022). Antibacterial, antiquorum sensing, antibiofilm activities and chemical profiling of selected South African medicinal plants against multi-drug resistant bacteria. *Journal of Medicinal Plants Research*.

[40] Truong D. H., Nguyen D. H., Ta N. T. A., Bui A. V., Do T. H., Nguyen H. C. (2019). Evaluation of the use of different solvents for phytochemical constituents, antioxidants, and *in vitro* anti-inflammatory activities of severinia buxifolia. *Journal of Food Quality*.

[41] Adam O. A. O., Abadi R. S. M., Ayoub S. M. H. (2019). The effect of extraction method and solvents on yield and antioxidant activity of certain Sudanese medicinal plant extracts. *The Journal of Phytopharmacology*.

[42] Eloff J. N., Angeh I. E., McGaw L. J. (2018). Solvent-solvent fractionation can increase the antifungal activity of a *Melianthus comosus* (*Melianthaceae*) acetone extract to yield a potentially useful commercial antifungal product. *Industrial Crops and Products*.

[43] Nile S. H., Park S. W. (2015). HPTLC densitometry method for simultaneous determination of flavonoids in selected medicinal plants. *Frontiers in Life Science*.

[44] Parasuraman S., Rao A., Balamurugan S., Muralidharan S., Jayaraj Kumar K., Vijayan V. (2014). An overview of liquid chromatography-mass spectroscopy instrumentation. *Pharmaceutical Methods*.

[45] Maroyi A. (2017). *Lippia javanica* (Burm.f.) spreng.: traditional and commercial uses and phytochemical and pharmacological significance in the African and Indian subcontinent. *Evidence-based Complementary and Alternative Medicine*.

[46] Kumar S., Pandey A. K. (2013). Chemistry and biological activities of flavonoids: an overview. *The Scientific World Journal*.

[47] Mohammad F. S., Patwekar M. F., Patwekar F. I. (2022). Are plant-derived flavonoids the emerging anti-coronavirus agents?. *INNOSC Theranostics and Pharmacological Sciences*.

[48] Patwekar F. I., Heroor S., Mohsina F. (2010). Evaluation of antimicrobial activity of *Tephrosia procumbens* buch ham. *Research Journal of Pharmacognosy and Phytochemistry*.

[49] Burt S. (2004). Essential oils: their antibacterial properties and potential applications in foods- A review. *International Journal of Food Microbiology*.

[50] Mahizan N. A., Yang S., Moo C.-L., Song A. A.-L. (2019). Terpene derivatives as a potential agent against antimicrobial resisant (AMR) pathogens. *Molecules*.

[51] Thapa D., Louis P., Losa R., Zweifel B., Wallace R. J. (2015). Essential oils have different effects on human pathogenic and commensal bacteria in mixed faecal fermentations compared with pure cultures. *Microbiology*.

[52] Shi C., Ma Y., Tian L. (2022). Verbascoside: an efficient and safe natural antibacterial adjuvant for preventing bacterial contamination of fresh meat. *Molecules*.

[53] Cosa S., Chaudhary S. K., Chen W., Combrinck S., Viljoen A. (2019). Exploring common culinary herbs and spices as potential anti-quorum sensing agents. *Nutrients*.

[54] Fils P., Cholley P., Gbaguidi-Haore H., Hocquet D., Sauget M., Bertrand X. (2021). ESBL-producing *Klebsiella pneumoniae* in a University hospital: molecular features, diffusion of epidemic clones and evaluation of cross-transmission. *PLoS One*.

[55] Londonkar R. L., Madire Kattegouga U., Shivsharanappa K., Hanchinalmath J. V. (2013). Phytochemical screening and *in vitro* antimicrobial activity of *Typha angustifolia* Linn leaves extract against pathogenic Gram negative micro organisms. *Journal of Pharmacy Research*.

[56] van Vuuren S., Muhlarhi T. (2017). Do South African medicinal plants used traditionally to treat infections respond differently to resistant microbial strains?. *South African Journal of Botany*.

[57] Mostafa A. A., Al-Askar A. A., Almaary K. S., Dawoud T. M., Sholkamy E. N., Bakri M. M. (2018). Antimicrobial activity of some plant extracts against bacterial strains causing food poisoning diseases. *Saudi Journal of Biological Sciences*.

[58] Perera M. M., Dighe S. N., Katavic P. L., Collet T. A. (2022). Antibacterial potential of extracts and phytoconstituents isolated from *Syncarpia hillii* leaves *in vitro*. *Plants*.

[59] Mogana R., Adhikari A., Tzar M. N., Ramliza R., Wiart C. (2020). Antibacterial activities of the extracts, fractions and isolated compounds from *Canarium patentinervium* miq. against bacterial clinical isolates. *BMC Complementary Medicine and Therapies*.

[60] Shriram V., Khare T., Bhagwat R., Shukla R., Kumar V. (2018). Inhibiting bacterial drug efflux pumps via phyto-therapeutics to combat threatening antimicrobial resistance. *Frontiers in Microbiology*.

[61] Oleksy-Wawrzyniak M., Junka A., Brożyna M. (2021). The *in vitro* ability of *Klebsiella pneumoniae* to form biofilm and the potential of various compounds to eradicate it from urinary catheters. *Pathogens*.

[62] Huang C., Tao S., Yuan J., Li X. (2022). Effect of sodium hypochlorite on biofilm of *Klebsiella pneumoniae* with different drug resistance. *American Journal of Infection Control*.

[63] Wang J., Liu Q., Dong D., Hu H., Wu B., Ren H. (2020). *In-situ* monitoring of the unstable bacterial adhesion process during wastewater biofilm formation: a comprehensive study. *Environment International*.

[64] Patil A., Munot N., Patwekar M. (2022). Encapsulation of lactic acid bacteria by lyophilisation with its effects on viability and adhesion properties. *Evidence-based Complementary and Alternative Medicine*.

[65] Ramos-Vivas J., Chapartegui-González I., Fernández-Martínez M. (2019). Biofilm formation by multidrug resistant *Enterobacteriaceae* strains isolated from solid organ transplant recipients. *Scientific Reports*.

[66] Shirinda H., Leonard C., Candy G., van Vuuren S. (2019). Antimicrobial activity and toxicity profile of selected southern African medicinal plants against neglected gut pathogens. *South African Journal of Science*.

[67] Jang M. H., Piao X. L., Kim J. M., Kwon S. W., Park J. H. (2008). Inhibition of cholinesterase and amyloid-*β* aggregation by resveratrol oligomers from *Vitis amurensis*. *Phytotherapy Research*.

[68] Liu Y., Han L., Yang H., Liu S., Huang C. (2020). Effect of apigenin on surface-associated characteristics and adherence of *Streptococcus mutans*. *Dental Materials Journal*.

[69] Bi Y., Xia G., Shi C. (2021). Therapeutic strategies against bacterial biofilms. *Fundamental Research*.

[70] Koo H., Yamada K. M. (2016). Dynamic cell-matrix interactions modulate microbial biofilm and tissue 3D microenvironments. *Current Opinion in Cell Biology*.

[71] Relucenti M., Familiari G., Donfrancesco O. (2021). Microscopy methods for biofilm imaging: focus on sem and VP-SEM pros and cons. *Biology*.

[72] Vyas N., Sammons R. L., Addison O., Dehghani H., Walmsley A. D. (2016). A quantitative method to measure biofilm removal efficiency from complex biomaterial surfaces using SEM and image analysis. *Scientific Reports*.

[73] Yamanaka T., Yamane K., Furukawa T. (2011). Comparison of the virulence of exopolysaccharide-producing *Prevotella intermedia* to exopolysaccharide non-producing periodontopathic organisms. *BMC Infectious Diseases*.

[74] Karygianni L., Ren Z., Koo H., Thurnheer T. (2020). Biofilm matrixome: extracellular components in structured microbial communities. *Trends in Microbiology*.

[75] Husain F. M., Ahmad I., Al-Thubiani A. S., Abulreesh H. H., AlHazza I. M., Aqil F. (2017). Leaf extracts of *Mangifera indica* L. inhibit quorum sensing regulated production of virulence factors and biofilm in test bacteria. *Frontiers in Microbiology*.

[76] Ansari M. J., Al-Ghamdi A., Usmani S. (2013). Effect of jujube honey on *Candida albicans* growth and biofilm formation. *Archives of Medical Research*.

[77] Pacheco T., Gomes A. E., Siqueira N. M. (2021). SdiA, a quorum-sensing regulator, suppresses fimbriae expression, biofilm formation, and quorum-sensing signaling molecules production in *Klebsiella pneumoniae*. *Frontiers in Microbiology*.

[78] Besharova O., Suchanek V. M., Hartmann R., Drescher K., Sourjik V. (2016). Diversification of gene expression during formation of static submerged biofilms by *Escherichia coli*. *Frontiers in Microbiology*.

[79] Anes J., Hurley D., Martins M., Fanning S. (2017). Exploring the genome and phenotype of multi-drug resistant *Klebsiella pneumoniae* of clinical origin. *Frontiers in Microbiology*.

[80] Pruteanu M., Hernández Lobato J. I., Stach T., Hengge R. (2020). Common plant flavonoids prevent the assembly of amyloid curli fibres and can interfere with bacterial biofilm formation. *Environmental Microbiology*.

[81] Yao B., Xiao X., Wang F., Zhou L., Zhang X., Zhang J. (2015). Clinical and molecular characteristics of multi-clone carbapenem-resistant hypervirulent (hypermucoviscous) *Klebsiella pneumoniae* isolates in a tertiary hospital in Beijing, China. *International Journal of Infectious Diseases*.

[82] Xu Q., Yang X., Chan E. W. C., Chen S. (2021). The hypermucoviscosity of hypervirulent *K. pneumoniae* confers the ability to evade neutrophil-mediated phagocytosis. *Virulence*.

[83] Lin Y. C., Lu M. C., Tang H. L. (2011). Assessment of hypermucoviscosity as a virulence factor for experimental *Klebsiella pneumoniae* infections: comparative virulence analysis with hypermucoviscosity-negative strain. *BMC Microbiology*.

[84] Shon A. S., Bajwa R. P., Russo T. A. (2013). Hypervirulent (hypermucoviscous) *Klebsiella pneumoniae*: a new and dangerous breed. *Virulence*.

[85] Patwekar M., Patwekar F., Alghamdi S. (2022). Vancomycin as an antibacterial agent capped with silver nanoparticles: an experimental potential analysis. *BioMed Research International*.

[86] Chen J., Luo X., Qiu H., Mackey V., Sun L., Ouyang X. (2018). Drug discovery and drug marketing with the critical roles of modern administration. *American Journal of Translational Research*.

