# Supplementary material for: Extracts of Selected South African Medicinal Plants Mitigate Virulence Factors in Multidrug-Resistant Strains of Klebsiella pneumoniae
Source: Evid Based Complement Alternat Med. 2023 Oct 14;2023:3146588. doi: 10.1155/2023/3146588 (PMC10590271; doi:10.1155/2023/3146588)
Supplement: Supplementary Materials — The following supplementary data form part of this manuscript which further provides relevant information on some of the findings obtained from this study. Figure S1 shows a standard curve which entails the regression equation (Y = 0.348X − 0.074) obtained for exopolysaccharide quantification. Here, Y stands for the absorbance derived from the unknown samples. Figure S2 illustrates the representative mass spectrometry chromatograms of the analysed plant extracts showing peaks that correspond to the data presented in Tables 2–4. [file 3146588.f1.pdf]

Extracts of selected South African medicinal plants mitigate virulence factors in multidrug resistant strains of *Klebsiella pneumoniae*

Idowu J. Adeosun[1], Itumeleng T. Baloyi[1], Sekelwa Cosa[1*]

[1] Division of Microbiology, Department of Biochemistry, Genetics and Microbiology, University of Pretoria, Private Bag X20, Hatfield Pretoria 0028, South Africa; u21747050@tuks.co.za (I.J.A.), u18372882@tuks.co.za (I.T.B.).

[*] **Corresponding author**: Sekelwa Cosa, Division of Microbiology, Department of Biochemistry, Genetics and Microbiology, University of Pretoria, Private Bag X20, Hatfield Pretoria 0028, South Africa. Tel: +27-12-420-4602, E-mail: sekelwa.cosa@up.ac.za

Supplementary materials

The following supplementary data forms part of this manuscript which further provides relevant information on some of the findings obtained from this study. **Figure S1**: shows a standard curve which entails the regression equation (Y = 0.348X - 0.074) obtained for exopolysaccharide quantification. Here, Y stands for the absorbance derived from the unknown samples. **Figure S2**: illustrates the representative mass spectrometry chromatograms of the analysed plant extracts showing peaks that correspond to the data presented in the manuscript (Tables 2-4).

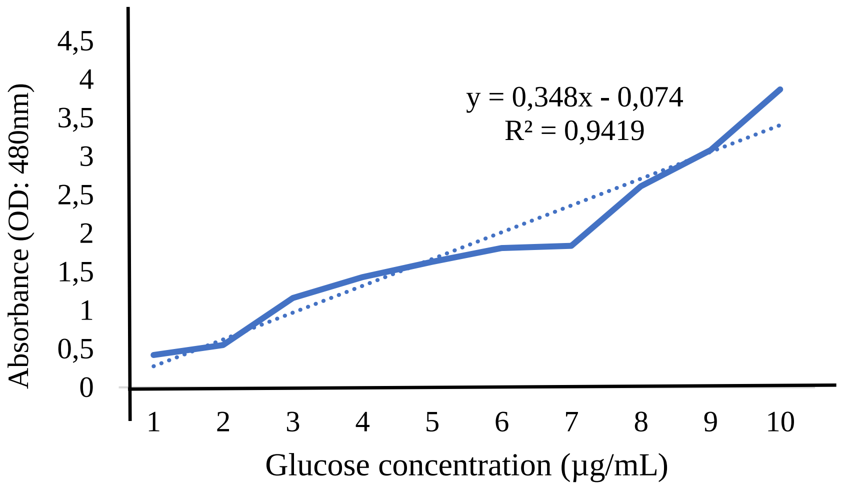

Figure S1: Standard curve showing the regression equation for EPS quantification.

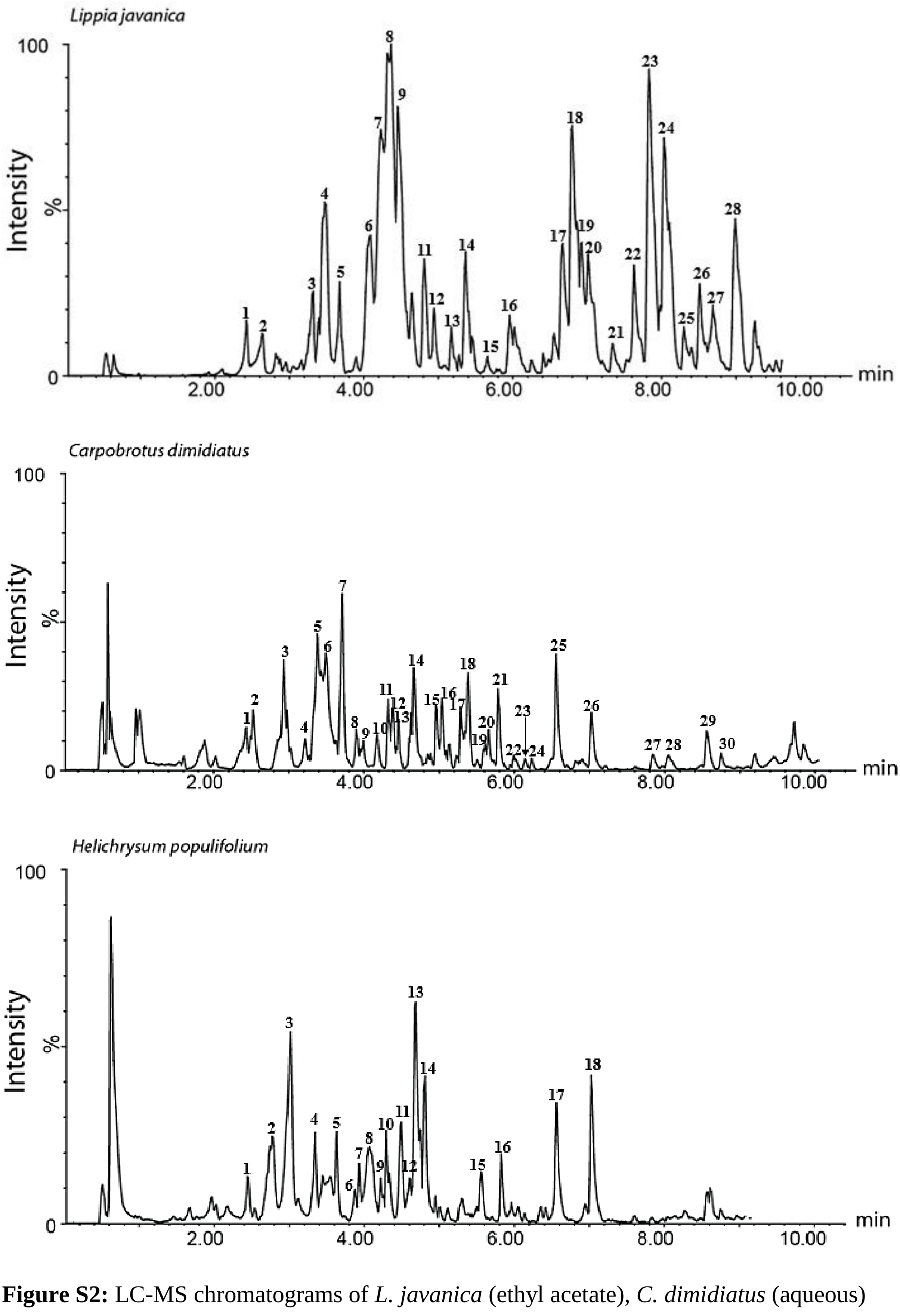

Figure S2: LC-MS chromatograms of *L. javanica* (ethyl acetate), *C. dimidiatus* (aqueous) and *H. populifolium* (aqueous) extract. All peaks correspond to the data presented in the manuscript (Tables 2-4).
